# High Burden of Co-Infection with Multiple Enteric Pathogens in Children Suffering with Diarrhoea from Rural and Peri-Urban Communities in South Africa

**DOI:** 10.3390/pathogens12020315

**Published:** 2023-02-14

**Authors:** Natasha Potgieter, Lee Heine, Jean Pierre Kabue Ngandu, Solanka Ellen Ledwaba, Tinyiko Zitha, Lutendo Sylvia Mudau, Piet Becker, Afsatou Ndama Traore, Tobias George Barnard

**Affiliations:** 1 One Health Research Group, Department of Biochemistry and Microbiology, Faculty of Science, Agriculture and Engineering, University of Venda, Thohoyandou 0950, Limpopo Province, South Africa; 2 Water and Health Research Centre, University of Johannesburg, Doornfontein 2094, Gauteng Province, South Africa; 3 Dept of Environmental Health, Faculty of Sciences, Tshwane University of Technology, Pretoria 0183, Gauteng Province, South Africa; 4 Research Office, Faculty of Health Sciences, University of Pretoria, Pretoria 0002, Gauteng Province, South Africa

**Keywords:** diarrhoea, infectious, pathogens, paediatric patients, South Africa, stool specimens

## Abstract

Infectious diarrhoea contributes to high morbidity and mortality in young children from sub-Saharan Africa. The aim of this study was to assess the prevalence of single and multiple diarrhoeal-causing pathogen combinations in children suffering from diarrhoea from rural and peri-urban communities in South Africa. A total of 275 diarrhoea stool specimens were collected between 2014 and 2016 from Hospitals and Primary Health Care clinics. The BioFire^®^ FilmArray^®^ Gastrointestinal panel was used to simultaneously detect 22 diarrhoea pathogens (viruses, bacteria, parasites) known to cause diarrhoea. A total of 82% (226/275) enteric pathogens were detected in the stool specimens. The two most detected bacterial, viral and parasitic pathogens each included: EAEC (42%), EPEC (32%), Adenovirus F40/41 (19%), Norovirus (15%), Giardia (8%) and Cryptosporidium (6%), respectively. Single enteric pathogen infections were recorded in 24% (65/275) specimens with *EAEC,* and *Norovirus* was found in 26% (17/65) and 14% (9/65) of the specimens, respectively. Multiple enteric pathogen combinations were recorded in 59% (161/275) of the stool specimens with 53% (85/161) containing two pathogens, 22% (35/161) containing three pathogens and 25% (41/161) containing four or more pathogens. The results from this study demonstrated the complex nature of pathogen co-infections in diarrhoeal episodes which could have an impact on treatment effectiveness.

## 1. Introduction

Diarrhoea is defined as passage of three or more unusually loose stool or watery stool of any frequency within 24 h [1]. The World Health Organisation (WHO) reports that almost 1.7 billion cases of childhood diarrhoeal diseases are recorded annually, with 525,000 children under the age of 5 years dying [2]. Sub-Saharan Africa and South Asia have the highest mortality rates, ranging from 50 to 150 per 100,000 people [3]. In Africa, 800,000 children die each year from diarrhoea and dehydration, which account for 25% to 75% of all childhood diseases, respectively [4,5]. In South Africa, the 2010 General Household Survey (GHS) showed that there were over 60,000 cases of childhood diarrhoea per month recorded in health care facilities, and approximately 9000 child diarrhoeal deaths in the same year [6].

The primary health care in South Africa is based on a system in which Primary Health Care clinics (PHC clinics) serve the rural and peri-urban communities, and nurses determine on the severity of the diarrhoea and whether it is necessary to refer the patient to the hospital where a medical doctor takes over the treatment [7,8]. Generally, children with diarrhoea in rural and peri-urban areas are treated with oral rehydration and/or antibiotic regimes without identifying the pathogen(s) responsible for infection. Thus, broad-spectrum antibiotics are overused, and bacterial resistance develops [9].

To improve clinical care and public health surveillance, it is important to correctly diagnose the aetiology of infectious diarrhoea [10]. A systematic literature review done in 2013 [11] on deaths due to diarrhoeal diseases, estimated that 70% deaths are attributable to 13 pathogens, of which Rotavirus, Enteropathogenic *Escherichia coli* (EPEC), Enterotoxigenic *Escherichia coli* (ETEC), Calicivirus and *Shigella* were the most important pathogens. The Global Enteric Multicenter Study (GEMS) agrees with this, but also includes *Cryptosporidium* spp. as an important pathogen [12]. The MAL-ED study [13,14] included the collection of 5399 stool specimens for surveillance purposes. Of these, 5160 (95.6%) were available for testing. However, only 3458 (64%) were included in the analysis of aetiologies of diarrhoeal episodes. Several geographical regions were included in the analyses, of which the Vhembe district of South Africa was one. Re-analysis of stool samples using probe-based qualitative PCR assays compartmentalised in TaqMan^®^ Array cards (Thermo Fisher, Waltham, MA, USA) for the detection of 29 enteric pathogens showed that for the Vhenda region in South Africa, the ten pathogens with the highest attributable incidence (from highest to lowest) were: *Shigella* spp., norovirus, sapovirus, *Campylobacter* spp., astrovirus, rotavirus, adenovirus, ETEC, tEPEC and *Cryptosporidium* spp. [13,14].

For public health interventions to be successful, it is important to determine the relative burden of pathogen-specific diarrhoea to compare the results to other studies with similar settings; to determine if the principal aetiologies of diarrhoea in the community are similar to those for more severe disease; and to understand the importance of mixed infection [15]. Although some diarrhoea cases have a single defined pathogen, most diarrhoea cases are thought to be caused by multiple pathogens [2]. Shrivastavha et al. [16] hypothesized that in cases with multiple infections, pathogens may lead to severe diarrhoea. The laboratories at referral hospitals in rural and peri-urban areas are not always able to perform standard tests on a regular basis [17]. Recent advances of molecular diagnostics tests that use multiplex assays able to target several different pathogens (bacteria, viruses and parasites) within a single reaction have made it possible to characterize the clinical aetiology and epidemiology of infectious diarrhoea [18,19,20,21,22]. In this study, the BioFire Filmarray Gastrointestinal (GI) panel, which detects 22 diarrhoeal pathogens, was used to determine the prevalence of single and multiple viral, bacterial and parasitic pathogens causing diarrhoea in children younger than five years of age from rural and peri-urban communities in South Africa to understand the aetiology of diarrhoea. Using this panel, the complex nature of co-infections could be assessed in treating diarrhoeal episodes. 

## 2. Materials and Methods

### 2.1. Patient Enrolment 

Only children who have lived in the Vhembe district for at least 7 days or more and experienced more than two watery/loose stools per day and have not taken any antibiotics during the week prior to providing a stool specimen were included in this study. 

### 2.2. Study Population and Stool Collection

The study focused on the Vhembe District Municipality in the northern part of the Limpopo province in South Africa, which comprises of four Local Municipalities, Musina, Mutale, Thulamela and Makhado. Two district hospitals (Louis Trichardt Memorial Hospital and Donald Fraser Hospital) and one regional hospital (Tshilidzini Hospital) were included in the study. Each of these hospitals have several Primary Health Care (PHC) clinics that serve under them and were included in this study for stool sample collection. Only severe cases of diarrhoea were referred by the nurses at the PHC clinics to the hospitals for further treatment. Both the Donald Fraser and Tshilidzini hospitals have clinics responsible for outpatient treatment.

Stool specimens were collected between 2014 and 2016 from rural and peri-urban PHC facilities and for five months during 2016 at the study hospitals from children under the age of five years suffering from diarrhoea as part of a larger study regarding the cost of treating severe diarrhoea. Nurses assisted in the collection of stool specimens, which were kept at 4 °C until collected and delivered to the laboratory where they were processed. 

### 2.3. Pathogen Detection

The commercially available BioFire^®^ Film Array^®^ GI Panel system was used per the manufacturer’s instructions (BioFire Diagnostics, Salt Lake City, UT, USA) to analyse all stool specimens. Briefly: only one specimen at a time was processed within a closed pouch where 200 μL of the stool specimen is added together with the supplied hydration solution. The system performs a nucleic acid extraction, multiplex PCR and melting analysis in 60 min and provides a printout of the results. The test simultaneously detects and identifies nucleic acids from the following 22 diarrhoea pathogens:

- **Bacteria**: Campylobacter [*C. jejuni*; *C. coli*; *C. upsaliansis*], *Clostridium difficile* [toxin A/B], *Plesiomonas shigelloides*, *Salmonella*, *V. cholerae*; *Vibrio* spp [*V. cholerae*; *V. parahaemolyticus*; *V. vulnificus*], *Yersinia enterocolitica*, Enteroaggregative *E. coli* (EAEC), Enteropathogenic *E. coli* (EPEC), Enterotoxigenic *E. coli* (ETEC [lt/st]), Shiga-like-toxin-producing *E. coli* [STEC stx1/stx2], *E. coli* O157, Shigella-EIEC.
- **Viruses**: Adenovirus F40/41, Sapovirus [I; II; IV; V], Astrovirus [HAstV 1-8], Norovirus [GI; GII], Rotavirus A.
- **Parasites**: Cryptosporidium, Cyclospora cayetanensis, Entamoeba histolytica, Giardia lamblia [*G. intestinalis*; *G. duodenalis*].

### 2.4. Statistical Analysis

All data were imported to a Microsoft Excel spreadsheet and analysed using the Strata 14 statistical package. The hospital samples were collected for only five months during 2016 and therefore descriptive data were used. Statistical analysis was reported using the Fischer’s exact test. 

## 3. Results 

### 3.1. Gender and Age Demographics of Study Group

A total of 275 children suffering from diarrhoea were part of the study and included 140 (52%) males and 128 (48%) females under the age of 5 years old. No statistical difference was seen between the male and female patients visiting the PHC clinics and the Hospitals (*p* = 0.303). No information on gender was recorded for seven of the patients during stool specimen collections (Table 1). 

The majority (80%; 221/275) of stool samples were collected from children aged between birth and 24 months. Only 265 stool specimens (96%) had data on the age of patient. The overall median age distribution for the study participants was 10 months [IQR 6 to 18 months], whereas the hospital cohort had the median age of 12 months [IQR 9 to 17 months] and the PHC clinic cohort had the median age of 9 months [IQR 4 to 18 months]. No statistical difference was observed between the age distribution of patients from the hospitals and PHC clinics (*p* = 0.293). Table 2 provides a breakdown of age distribution and frequency of stools received by age group and health facility.

### 3.2. Symptoms Reported in Study Cohort

**Figure 1:**
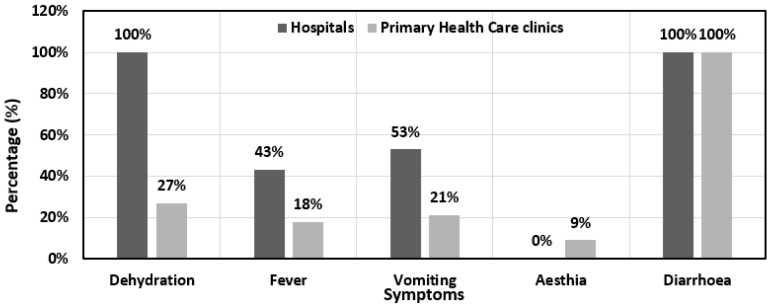
Distribution by recruitment site of clinical symptoms displayed by patients.

In the study cohort, 33% (90/275) of the patients recorded only one symptom, while 67% (185/275) of patients experienced multiple symptoms. All children (100%; 91/91) from the hospital group had multiple symptoms, whereas 49% (90/184) and 51% (95/184) of the children in the PHC clinic group had single and multiple symptoms, respectively. This was statistically significant (*p* = 0.00). The symptoms recorded for each patient on the questionnaire included dehydration, fever, vomiting, diarrhoea, and aesthia/listlessness (Figure 1).

### 3.3. Pathogen Prevalence in Stool Samples

From June 2014 to June 2016, 184 stool samples were collected from PHC clinics serving rural and peri-urban communities, with 78% (144/184) being positive for one or more pathogens. Viruses were found in 27% of the samples, parasites in 2% of the samples and bacteria in 69% of the samples. Between August and December 2016, 91 stool samples were collected from the three study hospitals, with 90% (82/91) of the samples being positive for one or more pathogens. Viruses were found in 59% of the samples, parasites in 5% of the samples and bacteria in 37% of the samples. Overall, a total of 82% (226/275) of the samples were positive for at least one pathogen in the GI panel; viruses were found in 11% (25/226), bacteria were detected in 17% (38/226) and parasites were detected in 1% (2/226) of the samples. 

### 3.4. Single vs. Multiple Infections

In total, 24% (65/275) of the stool specimens were positive for only one pathogen (Table 3). Overall, single infections were detected in 23% (43/184) of the clinic patient specimens and in 24% (22/91) of the hospital patient specimens. The most prevalent pathogens responsible for single infections were EAEC in 26% (17/65), Norovirus GI/GII in 14% (9/65), *Shigella*-EIEC in 12% (8/65), ETEC in 11% (7/65) and Adenovirus F40/41 in 11% (7/65) of the stools. A total of 59% (161/275) of the study cohort were infected with multiple pathogens (Table 3). Multiple or co-infections were detected in 55% (101/184) of the stool specimens from the clinic patients and 66% (60/91) were detected in the stool specimens from the hospital patients.

Table 4 indicates that bacteria/bacteria combinations were detected in 29% (46/161) of stool specimens; bacteria/parasite combinations were detected in 14% (23/161) of stool specimens; bacteria/virus/parasite combinations were detected in 6% (9/161) of stool specimens; virus/virus combinations were detected in 3% (5/161) of stool specimens; bacteria/virus combinations were detected in 47% (76/161) of stool specimens; virus/parasite and parasite/parasite combinations were detected in 1% (1/161) of stool specimens. Two pathogens were detected in 53% (85/161) of stool specimens; three pathogens were detected in 22% (35/161) of stool specimens; and four or more pathogens were detected in 25% (41/161) of stool specimens.

EPEC/EAEC combination was the highest mixed infection, detected in 5% (8/161) of the specimens. ETEC/EAEC combination was detected in 4.4% (7/161) of the specimens. EPEC/EAEC/Noro [GI/GII] combination was detected in 3.7% (6/161) of the specimens. Combinations of either ETEC/EPEC or EAEC/Rotavirus A or EAEC/Adenovirus F 40/41 or EAEC/Cryptosporidium were detected in 2.5% (4/161) of the stool specimens, respectively.

## 4. Discussion

The objective of this study was to show the prevalence of single and multiple diarrhoeal pathogen combinations in stool specimens collected from children under the age of five from rural and peri-urban communities. Several studies have reported that children in rural and peri-urban communities are exposed to enteric pathogens through a variety of transmission pathways including contaminated food, water, exposure to soil contaminated with animal faeces and through person-to-person contact [23,24,25,26]. 

The results in this study have shown that most stool specimens positive for diarrhoeagenic pathogens were collected from children aged between 0–24 months (Table 3), which correlates with other previously reported studies in the Limpopo province of South Africa [27,28]. 

Diarrhoeagenic *E. coli* strains have been frequently identified as a predominant cause of diarrhoea in children under the age of five years. In Mozambique, a study reported diarrhoeagenic *E. coli* as the cause of 42% of diarrhoea cases [29]. In Tanzania, a study showed that diarrhoeagenic *E. coli* was responsible for 70% of all diarrhoea cases [30]. In the present study, diarrhoeagenic *E. coli* strains were highly detected in single or mixed infections with other bacterial strains, viruses or parasites (Table 3, Table 4 and Table 5).

However, several studies in Africa have also indicated a high carriage level of diarrhoeagenic *E. coli* detection in asymptomatic cases [31,32]. In this study, EPEC was detected in 32% of the participants. EPEC are important in paediatric endemic diarrhoea and diarrhoea outbreaks [33]; atypical EPEC has been reported in association with prolonged diarrhoea [34] and has also been demonstrated to be more prevalent than typical EPEC in both developed and developing countries [33]. Nguyen et al. [35] have reported that diarrhoea caused by atypical EPEC is usually mild and generally not associated with dehydration; its importance lies in its association with prolonged diarrhoea, which is a major contributor to childhood illness in developing countries. In this study, ETEC was found in 22% of the participants. ETEC is a multivalent pathogen that causes recurrent infections that may adversely affect the nutritional status of children younger than two years of age and the susceptibility of infants and young children due to poor public health and hygiene conditions [36,37]. In this study, EAEC was the highest detected pathogen, in 42% of the participants. EAEC has been linked with persistent diarrhoea in children living in areas where EAEC is endemic [38]. Contaminated food appears to be the main source of EAEC infection and has been implicated in several foodborne outbreaks of diarrhoea [39,40]. The MAL-ED study [41] has shown the importance of subclinical EAEC infection and co-infections in children younger than two years of age and emphasized the importance of adequate maternal and child care, hygiene, sanitation and socio-economic factors.

High proportions of co-infections have been reported in low-income countries, where children may be exposed simultaneously to multiple enteric pathogens due to poor sanitary conditions [25,42,43]. Co-infections are a risk in persistent diarrhoea lasting longer than 14 days, which leads to death in young children [44]. Mixed or co-infections in Africa are common and have been reported by several studies [28,32,45]. From the results (Table 4), we did see co-infections of virus–bacteria–parasites combinations in 9 stool samples; co-infections of virus–parasite combinations in 1 stool sample; co-infections of different bacterial strain combinations in 46 stool samples; co-infections of bacteria–parasite combinations in 23 stool samples; co-infections of bacteria–virus combinations in 76 stool samples; and only 1 stool sample with different parasite (Giardia and Cryptosporidium) combinations. However, it may be difficult to indicate the exact role of each pathogen and to interpret if mechanisms of synergic enhancement of disease severity may be enacted.

Nyholm et al. [46] reported that Diarrhoeagenic *E. coli* can acquire virulence genes from other pathogroups via horizontal gene transfer, resulting in the development of what is termed intermediate pathogroups [47], or also referred to as hybrid [48] or blended virulence profiles and virulence combination [49]. The combinations of EHEC/EAEC from Germany [50] and STEC/ETEC from Finland are *E. coli* hybrid strains that have been reported to cause severe diarrhoea in humans [46]. Other *E. coli* combinations that have also been reported include EPEC/EAEC [51] and the *E. coli* O104:H4 strain in Germany, which possesses EAEC- and STEC-associated virulence genes [48]. This explains both the possibility of the emergence of extremely pathogenic strains [52] as well as the limitations of molecular techniques in distinguishing these strains from others in mixed cultures. More researchers are looking at the complex mechanisms of host–microbe and microbe–microbe interactions in the gastrointestinal tract, and details regarding the complex mechanisms of action and interaction are emerging [53,54]. 

Viruses such as *Adenovirus F40/41, Sapovirus [I; II; IV; V], Astrovirus [HAstV 1-8], Norovirus [GI; GII]* and *Rotavirus A* were all detected in the stool samples as either single or co-infections (Table 3, Table 4 and Table 5). Kotlofff et al. [12] conducted a multi-centre case-control study in Africa and South Asia and found that Rotavirus and, to a lesser extent, Adenovirus 40/41 (<5%) were the most common viruses causing moderate-to-severe diarrhoea in children. In the present study, Norovirus and Adenoviruses were the most frequent viruses isolated from the stool specimens, whereas Rotaviruses, Sapoviruses and Astroviruses were detected in low percentages (Table 4 and Table 5). Positive results from patients for adenovirus (17.8%) were higher compared to other studies conducted in other African countries: 10.4% in Tunisia [55], 8.0% in Senegal [56], 2.9% in Mozambique [57], 5.7% in the Central African Republic [58] and 3.8% in Angola [59]. Similarly, more patients tested positive for norovirus (14.7%) in the current study compared to: 4.2% in Mozambique [57] and 9.9% in the Central African Republic [58]. Detection rates of astrovirus in the present study (2.5%) were like those in studies conducted by Nhampossa et al. [57] and Gasparinho et al. [59] in Mozambique (1.7%) and Angola (2.6%), respectively. According to GEMS, Sapovirus prevalence, along with other enteric pathogens, has been linked with moderate to severe diarrhoea in developing countries [12]. Sapovirus infections were found in 36.6% (15/41) of African studies, with the GI and GII subtypes being more prevalent [60]. Detection rates of Sapovirus ranged between 0.8% to 18% in Burkina Faso, with studies in South Africa reporting prevalence of 4.1% and 7.7%, respectively [60].

Astroviruses in this study were found in three stool samples, as well as in combination with either EPEC-EAEC and EPEC-Shigella/EIEC, respectively (Table 4 and Table 5). In South Africa, Astroviruses have been frequently associated with Rotavirus infections in children [61]. Finkbeiner et al. [62] have shown a diversity of Astroviruses present in stool specimens from diarrhoea patients and have further speculated that there might be more unrecognized Astroviruses responsible for diarrhoea cases. Although no further classification of the Norovirus isolates in this study was performed, a previous study on Norovirus strains circulating in the Vhembe district in 2017 found that the GII subtype is more prevalent than the GI subtype in children under five years of age [63]. The presence of Rotavirus A in some stool samples in the present study might have been due to recombinant vaccine strains, which have been shown to shed in stool samples for up to eight weeks following inoculation [64]. Madhi et al. [65] and Groome et al. [66] have shown that the administration of Rotavirus vaccination treatment decreased the incidence of diarrhoea caused by the virus in South Africa. Other studies have also reported that there is an increase in the frequency of Norovirus as a cause of acute gastroenteritis in countries where the rotavirus vaccine has been introduced [67,68,69,70]. 

A recent study by Ikeda et al. [71] examining seasonal factors in relation to hospital-admitted diarrhoea cases in South Africa reported that higher cases of diarrhoea were observed during the warmer season than in the usual conditions in the dry winter season when temperatures range between 5 to 10 °C, and that dry conditions were more associated with diarrhoea in children younger than five years of age. The dry conditions in rural areas can lead to increased water storage which then increases the risk of water contamination, as well as less water accessible for personal hygiene, which increases the risk of potential diseases [2,26]. Virus detection may have been influenced by seasonality; Sapovirus and Rotavirus are more prevalent in cold months [72,73], while Astrovirus infections are more likely to occur in spring and summer [74]. In the current study, Norovirus was more prevalent in specimens collected from patients presenting with diarrhoea during the rainy season, and this is consistent with previous studies [75,76]. Seasonal and other environmental variations appear to have no influence on the prevalence of Adenovirus infection [77,78]. 

Only *Cryptosporidium* (13%) and *Giardia lamblia* (8%) parasites were detected in the stool samples. According to Omoladi et al. [79], the overall prevalence of *Cryptosporidium* spp infection in Southern African countries was 16.8%, which is mainly due to the high number of immunocompromised individuals in the region suffering from HIV/Aids. In this study, no samples tested positive for *Entamoema histolytica* or *Cyclospora cayetanensis.* However, Samie et al. [80] have previously reported on *E. histolytica* infections in the Vhembe region to be associated with diarrhoea in both healthy and immunocompromised patients. Also, in the Venda region, relatively low percentage of study participants testing positive for *C. cayetanensis* have been observed [81].

In a study conducted by Walker et al. [82], paired stool specimens were collected from 117 children under the age of five with acute, non-bloody, community-acquired diarrhoea who were admitted to one of four hospitals in Botswana. This study also used the BioFire^®^ Film Array^®^ (BioFire Diagnostics, Salt Lake City, UT, USA), and when the Walker et al. [82] study results were compared to the present study, similar rates of detection were noted for EAEC (42.7%), EPEC (41.0%), ETEC (17.9%), G. lamblia (9.4%), sapovirus (6.8%), *Cryptosporidium* spp. (6.0%), astrovirus (1.7%), *Salmonella* spp. (0.9%), *C. cayetanensis* (0.0%), *E. histolytica* (0.0%), *Y. enterocolitica* (0.0%) [82]. Detection rates were higher in participants in the Walker study for *Campylobacter* spp. (17.1%), *C. difficile* (7.7%), *V. cholerae* (2.6%) and rotavirus, which was detected in over half (52.1%) of the study participants, compared with only 12.9% in the present study [82]. Compared to the present study, detection rates were lower in participants in the Walker study for *P. shigelloides,* STEC, *Shigella*/EIEC (13.7%), adenovirus (10.3%) and norovirus (9.4%) [82].

Limitations of the study included: (1) PHC clinic specimens were collected over a period of 24 months from various clinics and then stored at −20 degrees; (2) Hospital specimens were the only specimens collected and tested within a short period of time; (3) Hospital specimens were only collected for 5 months in the study; (4) Participation in the study was voluntary, and so not all children presenting with diarrhoea at the respective health care facilities included in the study were recruited; (5) If the PHC clinic nurses experienced high patient volumes, the required time for specimen collection was not available and thus not all patients under the age of five presenting with diarrhoea at the clinic could be included in the study; (6) no asymptomatic stools were collected or tested; (7) the BioFire^®^ Film Array^®^ GI Panel (BioFire Diagnostics, Salt Lake City, UT, USA) does not distinguish between active infection and asymptomatic colonisation of the patient by potential pathogens [83]. Non-viable DNA and/or RNA may also be detected during analysis and could be misinterpreted as a true positive [83]. Further complicating the interpretation of results obtained by utilisation of the BioFire^®^ Film Array^®^ GI Panel (BioFire Diagnostics, Salt Lake City, UT, USA) is that several enteric pathogens are shed over a prolonged period by patients who have already recovered from an episode of diarrhoea and are asymptomatic at the time of testing [19]. Diagnoses should therefore be made by clinicians by interpreting laboratory results in conjunction with the clinical presentations of diarrhoea cases and the symptoms reported by patients. The rates of detection of pathogens in frozen versus fresh stool specimens using the BioFire^®^ (BioFire Diagnostics, Salt Lake City, UT, USA) system has not yet been published and thus it is unclear if the use of frozen specimens influenced the results obtained in the present study. The diversity of diarrhoeal pathogens detected by the BioFire^®^ Film Array^®^ panel in this study allows for the collection of useful data on the prevalence of diarrhoeal pathogens and co-infections. 

## 5. Conclusions

The simultaneous detection of a wide variety of pathogens in individual patients presenting with diarrhoea is becoming increasingly important as co-infection with multiple microbes has been shown to increase both morbidity and mortality even though the exact interactions of these organisms during pathogenesis has yet to be fully elucidated. Results of the current study reveal that diarrhoeagenic *E. coli* (specifically EAEC) are the predominant cause of diarrhoea in the population group tested in the Vhembe district, South Africa. The detection of such a high proportion of multiple enteric pathogens in mixed infections in the current study raises public health concerns. The results from this study also demonstrates the need for continuous epidemiological monitoring and surveillance of antimicrobial resistance to ensure correct treatment of acute and persistent diarrhoea. The data will guide the Department of Health in the patient treatment and management of diarrhoea, especially in outbreak situations or in critically ill patients. 

## Figures and Tables

**Table 1:** Gender distribution per recruitment site.

Gender	Health Facility	Frequency (%)	* Total (%)
Male	Hospitals	52 (57%)	140 (52%)
	Primary Health Care clinics	88 (50%)	
Female	Hospitals	39 (43%)	128 (48%)
	Primary Health Care clinics	89 (50%)	

* 7 stool samples had no information on gender.

**Table 2:** Age distribution of patients by recruitment site.

Age (Months)	Healthcare Facility	
	Hospitals [Frequency (%)]	Primary Health Care facilities [Frequency (%)]
Birth—11	55	60
12–23	30	22
24–36	4	6
37–48	10	6
49–60	1	5

**Table 3:** Single infections detected in the stool specimens (*n* = 65).

Pathogen Detected	Hospital Patients (*n*)	PHC Clinic Patients (*n*)	Total Patients (*n*)
**Bacteria infection**	**8**	**30**	**38**
Campylobacter [*jejuni, coli, upsaliansis*]	-	1	1
EAEC	4	13	17
EPEC	2	5	7
ETEC [lt/st]	-	4	4
STEC [stx1, stx2]	-	1	1
*Shigella*/EIEC	2	6	8
**Virus infection**	**13**	**12**	**25**
Adenovirus F40/41	2	5	7
Astrovirus	-	3	3
Norovirus [GI/GII]	8	1	9
Rotavirus A	2	3	5
Sapovirus [I, II, IV, V]	1	-	1
**Parasite infection**	**1**	**1**	**2**
Giardia lamblia [*intestinalis, duodenalis*]	1	-	1
Cryptosporidium	-	1	1
**Total:**	**22 (24%)**	**43 (23%)**	**65 (24%)**

**Table 4:** Multiple infections of diarrhoea-causing pathogens detected in stool samples (*n* = 161).

**Bacteria—Bacteria combinations (*n* = 46):** *E. coli* 0157—STEC—EAEC (*n* = 1) *E. coli* 0157—STEC (*n* = 1) EAEC—Campylobacter (*n* = 1) EAEC—Clostridium difficile toxin A/B (*n* = 1) EAEC—EPEC—Campylobacter (*n* = 3) EAEC—Salmonella (*n* = 1) EPEC—Campylobacter (*n* = 2) EPEC—Clostridium difficile toxin A/B (*n* = 1) EPEC—EAEC (*n* = 8) ETEC—EAEC (*n* = 7) ETEC—EPEC—Campylobacter (*n* = 1) ETEC—EPEC—EAEC (*n* = 1) ETEC—EPEC (*n* = 4) *Shigella*/EIEC—Campylobacter (*n* = 1) *Shigella*/EIEC—EPEC—EAEC (*n* = 1) *Shigella*/EIEC—EPEC (*n* = 2) *Shigella*/EIEC—ETEC—EAEC—Campylobacter (*n* = 1) *Shigella*/EIEC—ETEC—EAEC (*n* = 2) *Shigella/EIEC—ETEC—EPEC—*Clostridium difficile toxin A/B—Campylobacter (*n* = 1) *Shigella*/EIEC—ETEC—EPEC—EAEC (*n* = 1) *Shigella*/EIEC—ETEC (*n* = 3) STEC—Campylobacter (*n* = 1) STEC—ETEC—EAEC*—Plesiomonas shigelloides* (*n* = 1) **Bacteria—Parasite combinations (*n* = 23):** EAEC—*Cryptosporidium parvum* (*n* = 4) EAEC—Giardia lamblia (*n* = 1) EPEC—*Cryptosporidium parvum* (*n* = 1) EPEC—EAEC—Giardia lamblia (*n* = 3) EPEC—EAEC—Salmonella*—Shigella*/EIEC—Campylobacter—Clostridium difficile toxin A/B*—Cryptosporidium parvum* (*n* = 1) EPEC—Giardia lamblia (*n* = 3) EPEC*—Shigella*/EIEC—*Giardia lamblia* (*n* = 1) ETEC—EAEC—*Campylobacter—Cryptosporidium parvum* (*n* = 1) ETEC—EAEC*—Cryptosporidium parvum* (*n* = 1) ETEC—EPEC—EAEC*—Cryptosporidium parvum* (*n* = 1) ETEC—EPEC—EAEC*—Giardia lamblia* (*n* = 1) ETEC—EPEC—*Shigella*/EIEC*—Cryptosporidium parvum* (*n* = 1) ETEC—*Giardia lamblia* (*n* = 1) ETEC—*Shigella/EIEC—Giardia lamblia* (*n* = 1) *Shigella*/EIEC*—Giardia lamblia* (*n* = 2) **Bacteria—Virus—Parasite combinations (*n* = 9):** EAEC—Rotavirus A*—Giardia lamblia* (*n* = 1) EPEC—Rotavirus A—*Giardia lamblia* (*n* = 1) EPEC—Adenovirus F40/41—Cryptosporidium parvum (*n* = 1) *E*TEC—Norovirus GI/GII—Campylobacter*—Cryptosporidium parvum* (*n* = 1) *Shigella*/EIEC—Norovirus GI/GII—*Giardia lamblia* (*n* = 1) *Shigella*/EIEC—Adenovirus F40/41—*Giardia lamblia* (*n* = 1) *Shigella*/EIEC—Rotavirus A—*Giardia lamblia* (*n* = 1) *Shigella*/EIEC—EPEC—Sapovirus—*Cryptosporidium parvum* (*n* = 1) *Shigella*/EIEC—EPEC—EAEC—Adenovirus F40/41—*Giardia lamblia* (*n* = 1) **Virus—Virus combinations (*n* = 5):** Rotavirus A—Adenovirus F40/41 (*n* = 3) Norovirus—Adenovirus F40/41 (*n* = 1) Rotavirus A—Norovirus—Adenovirus F40/41 (*n* = 1) **Parasite—Parasite combinations (*n* = 1):** *Giardia lamblia—Cryptosporidium parvum* (*n* = 1)	**Bacteria—Virus combinations (*n* = 76):** EAEC—Adenovirus F40/41—Rotavirus A (*n* = 1) EAEC—Adenovirus F40/41 (*n* = 4) EAEC*—Campylobacter—*Norovirus GI/GII (*n* = 1) EAEC—Norovirus GI/GII (*n* = 3) EAEC—Rotavirus A (*n* = 4) EAEC—Sapovirus (*n* = 1) EAEC*—Shigella/E*IEC—Adenovirus F40/41 (*n* = 1) EAEC—*Shigella*/EIEC—Sapovirus (*n* = 1) EAEC—STEC—Adenovirus F40/41 (*n* = 1) EAEC—STEC—*Shigella*/EIEC*—Salmonella—*Adenovirus F40/41 (*n* = 1) EPEC—*Clostridium difficile toxin A/B—*Norovirus GI/GII (*n* = 2) EPEC—EAEC—Adenovirus F40/41 (*n* = 1) EPEC—EAEC—Astrovirus (*n* = 1) EPEC—EAEC*—Clostridium difficile toxin A/B—*Norovirus GI/GII—Adenovirus F40/41 (*n* = 1) EPEC—EAEC—Norovirus GI/GII (*n* = 6) EPEC—EAEC—Rotavirus A—Norovirus GI/GII (*n* = 1) EPEC—EAEC*—Shigella*/EIEC—Adenovirus F40/41 (*n* = 2) EPEC—EAEC—*Shigella*/EIEC—Campylobacter—Adenovirus F40/41 (*n* = 1) *EPEC—EAEC—Shigella/EIEC—Norovirus GI/GII—*Adenovirus F40/41 (*n* = 1) EPEC—EAEC—*Shigella*/EIEC—Norovirus GI/GII—Adenovirus F40/41—Rotavirus A (*n* = 1) EPEC—EAEC—*Shigella*/EIEC—Norovirus GI/GII (*n* = 1) EPEC—EAEC—*Shigella*/EIEC—Rotavirus A (*n* = 1) EPEC—ETEC—EAEC—Adenovirus F40/41(*n* = 1) EPEC—ETEC—EAEC—Adenovirus F40/41—Rotavirus A (*n* = 1) EPEC—ETEC—EAEC—Norovirus GI/GII (*n* = 1) EPEC—ETEC—EAEC—Sapovirus (*n* = 1) EPEC—Norovirus GI/GII (*n* = 2) EPEC—Rotavirus A (*n* = 3) EPEC—*Salmonella—*Rotavirus A (*n* = 1) EPEC—*Shigella*/EIEC—Astrovirus (*n* = 1) ETEC—Adenovirus F40/41 (*n* = 1) ETEC—EAEC—Rotavirus A—Adenovirus F40/41 (*n* = 1) ETEC—EAEC—Rotavirus A (*n* = 1) ETEC—EAEC—Sapovirus—Adenovirus F40/41 (*n* = 1) ETEC—EAEC—*Shigella*/EIEC—Norovirus GI/GII—Rotavirus A (*n* = 1) ETEC—EAEC*—Shigella*/EIEC—Norovirus GI/GII (*n* = 1) ETEC—EAEC*—Shigella*/EIEC*—Plesiomonas shigelloides*—Norovirus GI/GII—Adenovirus F40/41—Rotavirus A (*n* = 1) ETEC—EAEC—STEC—Campylobacter—Adenovirus F40/41 (*n* = 1) ETEC—EAEC—STEC—*Shigella*/EIEC—Adenovirus F40/41 (*n* = 1) ETEC—EPEC—EAEC—Campylobacter*—Shigella*/EIEC—Adenovirus F40/41 (*n* = 1) ETEC—EPEC—EAEC—Campylobacter—*Shigella*/EIEC—Norovirus GI/GII (*n* = 1) ETEC—EPEC—EAEC—Campylobacter -Norovirus GI/GII (*n* = 1) ETEC—EPEC—EAEC—*Shigella*/EIEC—Adenovirus F40/41—Norovirus GI/GII (*n* = 1) ETEC—EPEC—EAEC—*Shigella*/EIEC—Adenovirus F40/41—Rotavirus A (*n* = 1) ETEC—EPEC—EAEC—*Shigella*/EIEC—Adenovirus F40/41 (*n* = 2) ETEC—EPEC—Rotavirus A (*n* = 1) ETEC—EPEC—*Shigella/E*IEC—Adenovirus F40/41 (*n* = 1) ETEC—Norovirus GI/GII (*n* = 3) Adenovirus F40/41—Campylobacter (*n* = 3) *Salmonella*—Sapovirus (*n* = 1) *Shigella*/EIEC—Adenovirus F40/41—Rotavirus A (*n* = 1) *Shigella*/EIEC—Adenovirus F40/41 (*n* = 1) *Shigella*/EIEC—Campylobacter—Adenovirus F40/41 (*n* = 1) **Virus—Parasite combinations (*n* = 1):** Sapovirus—Adenovirus F40/41*—Giardia lamblia* (*n* = 1)

**Table 5:** Summary of diarrhoeal pathogens detected in stool samples ordered from most to least prevalent.

Diarrhoea Pathogen	Study Cohort % Positive (*n*) [95% Confidence Interval]	Pathogen Distribution in Stool Samples per Health Care Facility		Pathogen Distribution in Stool Samples per Age Group [Only Age Information for 265 Stool Samples]				
		Hospital Cohort % Positive (*n*)	PHC Clinic Cohort % Positive (*n*)	≤12 Months	13–24 Months	25–36 Months	37–48 Months	49–60 Months
EAEC	42 (115/275) [*35.92–47.89*]	49 (45/91)	38 (70/184)	69	24	5	12	1
EPEC	32 (88/275) [*26.52–36.87*]	37 (34/91)	29 (54/184)	48	26	2	8	2
ETEC [lt/st]	21 (59/275) [*16.75–26.78*]	24 (22/91)	20 (37/184)	26	16	5	10	-
Shigella-EIEC	20 (55/275) [*15.43–25.22*]	19 (17/91)	21 (38/184)	25	15	5	7	2
Adenovirus F40/41	19 (51/275) [*14.13–23.65*]	14 (13/91)	21 (38/184)	36	9	2	4	-
Norovirus [GI/GII]	15 (42/275) [*11.23–20.08*]	32 (29/91)	7 (13/184)	24	13	1	2	-
Rotavirus A	13 (36/275) [*9.34–17.66*]	10 (9/91)	15 (27/184)	16	11	-	5	2
Campylobacter [*jejuni, coli, upsaliansis*]	9 (25/275) [*5.96–13.12*]	13 (12/91)	7 (13/184)	19	4	1	1	-
Giardia lamblia [*intestinalis, duodenalis*]	8 (21/275) [*4.79–11.44*]	8 (7/91)	8 (14/184)	7	6	1	2	3
Cryptosporidium	6 (16/275) [*3.36–9.27*]	11 (10/91)	3 (6/184)	7	8	1	-	-
Sapovirus [I, II, IV, V]	3 (9/275) [*1.40–6.12*]	2 (2/91)	4 (7/184)	4	2	2	1	-
STEC [stx1, stex2]	3 (9/275) [*1.50–6.12*]	2 (2/91)	4 (7/184)	4	2	-	2	-
*Clostridium difficile* *toxin A/B*	3 (7/275) [*1.03–5.17*]	3 (3/91)	2 (4/184)	6	-	1	-	-
Astrovirus	2 (5/275) [*0.59–4.19*]	1 (1/91)	2 (4/184)	3	2	-	-	-
Salmonella	2 (5/275) [*0.59–4.19*]	3 (3/91)	1 (2/184)	5	-	-	-	-
*Plesiomonas shigelloides*	1 (2/275) [*0.09–2.60*]	2 (2/91)	0 (0/184)	1	-	-	1	-
*E. coli* 0157	1 (2/275) [*0.09–2.60*]	0 (0/91)	1 (2/184)	-	1	-	-	-
Single infections (see Table 3)	24 (65/275)	24 (22/91)	23 (43/184)	39	14	4	5	3
Multiple infections (see Table 4)	59 (161/275)	66 (60/91)	55 (101/184)	86	43	9	14	3

## Data Availability

The data presented in this study are available on request from the corresponding author. The data are not publicly available due to protection of patients.
